# Supplementary material for: Unveiling the Bioactive Potential of the Invasive Jellyfish Phyllorhiza punctata Through Integrative Transcriptomic and Proteomic Analyses
Source: Biomolecules. 2025 Aug 4;15(8):1121. doi: 10.3390/biom15081121 (PMC12383608; doi:10.3390/biom15081121)
Supplement: Supplementary file 1 [file biomolecules-15-01121-s001.zip › Suplementary Material/FileS3/PEPFOLD4/XP_065051250_1006/clusters.html]

 PEP-FOLD clusters


Download archive of all models  here   

|  |  |  |
| --- | --- | --- |
| # | file | sOPEP |
| # cluster 1 - Size: 99 |  |  |
|  | XP\_065051250\_1006-model1 | -19.3454 |
|  | XP\_065051250\_1006-model1.1 | -19.3016 |
|  | XP\_065051250\_1006-model1.2 | -19.3016 |
|  | XP\_065051250\_1006-model1.3 | -19.3016 |
|  | XP\_065051250\_1006-model1.4 | -19.2574 |
|  | XP\_065051250\_1006-model1.5 | -19.2509 |
|  | XP\_065051250\_1006-model1.6 | -19.2509 |
|  | XP\_065051250\_1006-model1.7 | -19.2509 |
|  | XP\_065051250\_1006-model1.8 | -19.2509 |
|  | XP\_065051250\_1006-model1.9 | -19.2509 |
|  | XP\_065051250\_1006-model1.10 | -19.2509 |
|  | XP\_065051250\_1006-model1.11 | -19.2509 |
|  | XP\_065051250\_1006-model1.12 | -19.2509 |
|  | XP\_065051250\_1006-model1.13 | -19.2509 |
|  | XP\_065051250\_1006-model1.14 | -19.2509 |
|  | XP\_065051250\_1006-model1.15 | -19.2509 |
|  | XP\_065051250\_1006-model1.16 | -19.2413 |
|  | XP\_065051250\_1006-model1.17 | -19.2413 |
|  | XP\_065051250\_1006-model1.18 | -19.2413 |
|  | XP\_065051250\_1006-model1.19 | -19.2413 |
|  | XP\_065051250\_1006-model1.20 | -19.2413 |
|  | XP\_065051250\_1006-model1.21 | -19.2413 |
|  | XP\_065051250\_1006-model1.22 | -19.2413 |
|  | XP\_065051250\_1006-model1.23 | -19.2413 |
|  | XP\_065051250\_1006-model1.24 | -19.2413 |
|  | XP\_065051250\_1006-model1.25 | -19.2413 |
|  | XP\_065051250\_1006-model1.26 | -19.2413 |
|  | XP\_065051250\_1006-model1.27 | -19.2413 |
|  | XP\_065051250\_1006-model1.28 | -19.2413 |
|  | XP\_065051250\_1006-model1.29 | -19.2413 |
|  | XP\_065051250\_1006-model1.30 | -19.2413 |
|  | XP\_065051250\_1006-model1.31 | -19.2413 |
|  | XP\_065051250\_1006-model1.32 | -19.2291 |
|  | XP\_065051250\_1006-model1.33 | -19.223 |
|  | XP\_065051250\_1006-model1.34 | -19.2225 |
|  | XP\_065051250\_1006-model1.35 | -19.2225 |
|  | XP\_065051250\_1006-model1.36 | -19.2225 |
|  | XP\_065051250\_1006-model1.37 | -19.2144 |
|  | XP\_065051250\_1006-model1.38 | -19.2144 |
|  | XP\_065051250\_1006-model1.39 | -19.1971 |
|  | XP\_065051250\_1006-model1.40 | -19.1733 |
|  | XP\_065051250\_1006-model1.41 | -19.1733 |
|  | XP\_065051250\_1006-model1.42 | -19.1733 |
|  | XP\_065051250\_1006-model1.43 | -19.1733 |
|  | XP\_065051250\_1006-model1.44 | -19.1733 |
|  | XP\_065051250\_1006-model1.45 | -19.1675 |
|  | XP\_065051250\_1006-model1.46 | -19.1643 |
|  | XP\_065051250\_1006-model1.47 | -19.1643 |
|  | XP\_065051250\_1006-model1.48 | -19.1635 |
|  | XP\_065051250\_1006-model1.49 | -19.1462 |
|  | XP\_065051250\_1006-model1.50 | -19.1402 |
|  | XP\_065051250\_1006-model1.51 | -19.1402 |
|  | XP\_065051250\_1006-model1.52 | -19.1319 |
|  | XP\_065051250\_1006-model1.53 | -19.1317 |
|  | XP\_065051250\_1006-model1.54 | -19.1219 |
|  | XP\_065051250\_1006-model1.55 | -19.1219 |
|  | XP\_065051250\_1006-model1.56 | -19.1219 |
|  | XP\_065051250\_1006-model1.57 | -19.1219 |
|  | XP\_065051250\_1006-model1.58 | -19.1074 |
|  | XP\_065051250\_1006-model1.59 | -19.1074 |
|  | XP\_065051250\_1006-model1.60 | -19.1074 |
|  | XP\_065051250\_1006-model1.61 | -19.1065 |
|  | XP\_065051250\_1006-model1.62 | -19.1065 |
|  | XP\_065051250\_1006-model1.63 | -19.1065 |
|  | XP\_065051250\_1006-model1.64 | -19.1065 |
|  | XP\_065051250\_1006-model1.65 | -19.1065 |
|  | XP\_065051250\_1006-model1.66 | -19.1065 |
|  | XP\_065051250\_1006-model1.67 | -19.1058 |
|  | XP\_065051250\_1006-model1.68 | -19.0996 |
|  | XP\_065051250\_1006-model1.69 | -19.0811 |
|  | XP\_065051250\_1006-model1.70 | -19.0743 |
|  | XP\_065051250\_1006-model1.71 | -19.0736 |
|  | XP\_065051250\_1006-model1.72 | -19.0708 |
|  | XP\_065051250\_1006-model1.73 | -19.0708 |
|  | XP\_065051250\_1006-model1.74 | -19.0583 |
|  | XP\_065051250\_1006-model1.75 | -19.0532 |
|  | XP\_065051250\_1006-model1.76 | -19.0432 |
|  | XP\_065051250\_1006-model1.77 | -19.0402 |
|  | XP\_065051250\_1006-model1.78 | -19.0395 |
|  | XP\_065051250\_1006-model1.79 | -19.0395 |
|  | XP\_065051250\_1006-model1.80 | -19.0381 |
|  | XP\_065051250\_1006-model1.81 | -19.0307 |
|  | XP\_065051250\_1006-model1.82 | -19.0307 |
|  | XP\_065051250\_1006-model1.83 | -19.0295 |
|  | XP\_065051250\_1006-model1.84 | -19.0259 |
|  | XP\_065051250\_1006-model1.85 | -19.0259 |
|  | XP\_065051250\_1006-model1.86 | -19.0196 |
|  | XP\_065051250\_1006-model1.87 | -19.0196 |
|  | XP\_065051250\_1006-model1.88 | -19.0095 |
|  | XP\_065051250\_1006-model1.89 | -19.0032 |
|  | XP\_065051250\_1006-model1.90 | -19.0014 |
|  | XP\_065051250\_1006-model1.91 | -18.994 |
|  | XP\_065051250\_1006-model1.92 | -18.9896 |
|  | XP\_065051250\_1006-model1.93 | -18.9854 |
|  | XP\_065051250\_1006-model1.94 | -18.9824 |
|  | XP\_065051250\_1006-model1.95 | -18.9718 |
|  | XP\_065051250\_1006-model1.96 | -18.9718 |
|  | XP\_065051250\_1006-model1.97 | -18.9388 |
|  | XP\_065051250\_1006-model1.98 | -18.9281 |
| # cluster 2 - Size: 1 |  |  |
|  | XP\_065051250\_1006-model2 | -10.9732 |
|  |  |  |
